# A Robust Post-Processing Workflow for Datasets with Motion Artifacts in Diffusion Kurtosis Imaging

**DOI:** 10.1371/journal.pone.0094592

**Published:** 2014-04-11

**Authors:** Xianjun Li, Jian Yang, Jie Gao, Xue Luo, Zhenyu Zhou, Yajie Hu, Ed X. Wu, Mingxi Wan

**Affiliations:** 1 Radiology Department of the First Affiliated Hospital, Xi'an Jiaotong University, Xi'an, Shaanxi, People's Republic of China; 2 Department of Biomedical Engineering, the Key Laboratory of Biomedical Information Engineering of the Ministry of Education, School of Life Science and Technology, Xi'an Jiaotong University, Xi'an, Shaanxi, People's Republic of China; 3 Laboratory of Biomedical Imaging and Signal Processing, the University of Hong Kong, Hong Kong SAR, People's Republic of China; Institute of Automation, Chinese Academy of Sciences, China

## Abstract

**Purpose:**

The aim of this study was to develop a robust post-processing workflow for motion-corrupted datasets in diffusion kurtosis imaging (DKI).

**Materials and methods:**

The proposed workflow consisted of brain extraction, rigid registration, distortion correction, artifacts rejection, spatial smoothing and tensor estimation. Rigid registration was utilized to correct misalignments. Motion artifacts were rejected by using local Pearson correlation coefficient (LPCC). The performance of LPCC in characterizing relative differences between artifacts and artifact-free images was compared with that of the conventional correlation coefficient in 10 randomly selected DKI datasets. The influence of rejected artifacts with information of gradient directions and b values for the parameter estimation was investigated by using mean square error (MSE). The variance of noise was used as the criterion for MSEs. The clinical practicality of the proposed workflow was evaluated by the image quality and measurements in regions of interest on 36 DKI datasets, including 18 artifact-free (18 pediatric subjects) and 18 motion-corrupted datasets (15 pediatric subjects and 3 essential tremor patients).

**Results:**

The relative difference between artifacts and artifact-free images calculated by LPCC was larger than that of the conventional correlation coefficient (p<0.05). It indicated that LPCC was more sensitive in detecting motion artifacts. MSEs of all derived parameters from the reserved data after the artifacts rejection were smaller than the variance of the noise. It suggested that influence of rejected artifacts was less than influence of noise on the precision of derived parameters. The proposed workflow improved the image quality and reduced the measurement biases significantly on motion-corrupted datasets (p<0.05).

**Conclusion:**

The proposed post-processing workflow was reliable to improve the image quality and the measurement precision of the derived parameters on motion-corrupted DKI datasets. The workflow provided an effective post-processing method for clinical applications of DKI in subjects with involuntary movements.

## Introduction

Motion artifacts not only increase the variability of measures but also introduce biases which may lead to false-positive findings [1]. The head motion must be concerned during the post-processing in the diffusion magnetic resonance imaging (MRI) techniques. As an extension of diffusion tensor imaging (DTI), diffusion kurtosis imaging (DKI) provides more specific information than DTI [2], [3]. Compared with the conventional DTI, multiple b values and more diffusion gradient directions per nonzero b value in DKI necessitate a longer scan time. During the acquisition of DKI datasets, the head motion is common, especially in the pediatric subjects and patients with involuntary movements. The inter-volume misalignment caused by the slight head motion may be adjusted by using registration methods [4], [5]. However, the severe signal loss caused by the sudden tissue displacement with a large amplitude during the diffusion MRI scanning cannot be recovered [1], [4]. Therefore, a post-processing workflow to correct motion-corrupted datasets is of great importance for the clinical application of DKI, also the same for DTI and high angular resolution diffusion imaging (HARDI), etc.

In the DTI post-processing procedure, three kinds of approaches for motion artifacts detection and rejection were used: voxel-wise, slice-wise and volume-wise strategies. The Geman-Mclure M-estimator (GMM) [6] and robust estimation of tensor by outlier rejection (RESTORE) [7] are typical voxel-wise methods. These methods estimate the tensor by using conventional techniques before the outlier rejection. Outlier voxels are determined based on the residuals of the fitted data to the raw data. The outlier is rejected by using a designed weighting function. For the corrupted datasets with large-scale motion, the registration between diffusion weighted images (DWIs) is difficult. Mismatching between slices is an unsolved problem in voxel-wise methods [8]. Slice-wise methods for artifacts detection and rejection are proposed based on signal intensity information [9], texture information [5], or the consistency of derived parameters [10]. Slice-wise techniques detect artifacts in certain gradient directions with slice by slice mode. In the artifacts detection method based on the consistency of derived parameters, iterative estimations of diffusion tensors are required, which limits its applications. The corrected inter-slice intensity discontinuity (cISID) [11] is a powerful method to characterize the intensity discontinuity of DWIs. However, the cISID may fail to detect consecutive artifacts. The cISID method must be combined with other robust fitting techniques to increase the detection stability [11]. The combination of local binary patterns (LBP) and two-dimension (2D) partial least squares (PLS) has been demonstrated for detecting artifacts reliably [5]. However, the extraction of texture information from thousands of DWIs in DKI is time-consuming. Normalized correlation coefficient (NCC) is an efficient method. The global image information in NCC may weaken its reliability in detecting local artifacts. The volume-wise method based on correlation coefficient may discard the entire volume when several slices were corrupted by motion [12]. However, the valid slices without motion artifacts may also be rejected because of the neighboring artifacts slices in the same volume. It is necessary to develop an effective artifacts rejection method for the post-processing of DKI datasets. Pearson correlation coefficient is used frequently for characterizing the agreement between images [13]. A modified Pearson correlation coefficient based on the local image information may be suitable for the artifacts detection on DKI datasets with motion artifacts.

In this study, we focused on the slice-wise method for the motion artifacts rejection and proposed a robust post-processing workflow for motion-corrupted DKI datasets. Local Pearson correlation coefficient (LPCC) was compared with the conventional correlation coefficient in detecting motion artifacts. The feasibility of the artifacts rejection for DKI was investigated by using the mean square error (MSE). Finally, the applicability of the proposed workflow was evaluated by the image quality and measurements in the region of interest (ROI) on 36 DKI datasets, including 18 artifact-free (18 pediatric subjects) and 18 motion-corrupted datasets (15 pediatric subjects and 3 patients with essential tremor).

## Materials and Methods

The study complied with institutional guidelines and regulations and was approved by the Ethics Committee of the First Affiliated Hospital, Xi'an Jiaotong University. Written informed consents were obtained from the adult subjects and the parents of the pediatric subjects.

### 2.1 Theory

**Figure 1:**
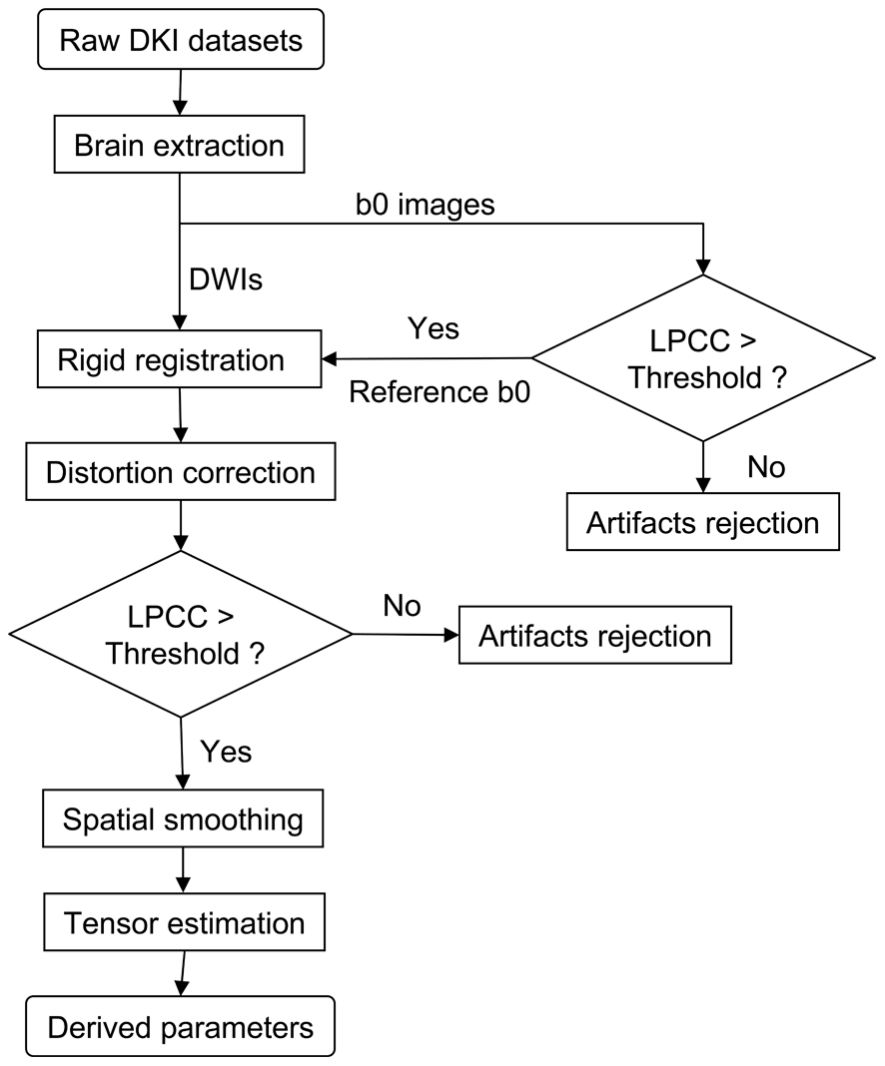
Programming flowchart of the robust post-processing workflow for DKI datasets with motion artifacts. DWIs: diffusion weighted images; LPCC: local Pearson correlation coefficient.

In the conventional post-processing workflow for DKI datasets, DWIs are smoothed by using a Gaussian kernel before the tensor estimation [14]. We proposed a robust post-processing workflow for the motion-corrupted datasets. As shown in Figure 1, the proposed workflow consisted of brain extraction, rigid registration, distortion correction, artifacts rejection, spatial smoothing and tensor estimation. Motion-related problems were concerned.

#### 2.1.1 Misalignment correction

The slight head motion during the MRI scan caused the inter-volume misalignment which could be solved by using registration methods [4], [5]. Rigid registration was used in the proposed post-processing workflow to correct the inter-volume misalignment.

#### 2.1.2 Motion artifacts rejection based on LPCC

The correlation between two images based on the signal intensity or brightness is a simple method to characterize the agreement between images [13]. As a conventional correlation coefficient, NCC was used for the artifacts rejection in the DTI quality control [9]. Local artifacts in DWIs may be drowned by the neighboring valid signals during the artifacts detection by using NCC. To obtain the local correlation information, one slice is divided into several sub-regions. The Pearson correlation coefficient (PCC) between the reference (b0) and the object slice is calculated region by region. Then PCCs are weighted to construct a combined coefficient, LPCC.

For the human brain DKI data, there are two parts in the DWIs after brain extraction: the tissue part and the background part. Therefore there are three kinds of sub-regions: regions containing the background (*S_b_*), regions containing tissues (*S_t_*), and regions containing both the background and tissues (*S_bt_*). Let *N_b_* denote the number of regions containing only the background. Weighting coefficients are defined as follows:
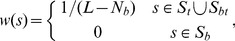
(1)where *L* is the number of sub-regions.

The LPCC is calculated as follows:
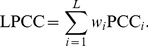
(2)


The motion artifacts could be detected and rejected by setting a defined threshold on the LPCCs.

### 2.2 Subjects and data acquisition

In this study, we scanned 36 subjects, including pediatric subjects (22 males and 11 females; age range: 4 days ∼6 years old; mean age  = 205 days old) and adult patients with the essential tremor from the neurology department (2 males and 1 female; age range: 30∼34 years old; mean age  = 32 years old). The parents of the kids were informed about the goals and risks involved in the MR scan. The kids were all sedated (oral chloral hydrate, 50 mg/kg) before the MRI scan. The neonates were laid in a supine position and snugly swaddled in blankets. A pediatrician, experienced in the resuscitation, was present during the MRI scan. Micro earplugs were prepared and placed in the bilateral external acoustic meatuses of the subjects for the hearing protection. The heads of the subjects were immobilized by the molded foam. The temperature was maintained and the heart rate and the oxygen saturation were monitored throughout the procedure.

A single short echo planar imaging sequence was performed for acquisition of DKI datasets by using an 8-channel phase array radio-frequency head coil in a 3 T scanner (Signa HDxt, General Electric Medical System, Milwaukee, WI, USA). DKI was carried out with the following variables: b values  = 0, 500, 1000, 1500, 2000 and 2500 s/mm^2^; 25 gradient directions per nonzero b value; NEX = 1; TR = 4000 ms; TE range: 106.6∼108.5 ms; 10 slices with slice thickness  = 5 mm; field of view  = 180×180 mm^2^ for neonates and infants, 180×180 mm^2^ or 240×240 mm^2^ for children according to their brain sizes, 240×240 mm^2^ for adults; matrix  = 128×128. The acquisition time was 8 minutes 44 seconds.

### 2.3 Data post-processing

#### 2.3.1 Brain extraction

Extracted brain images were acquired by using the Brain Extraction Tool (BET), the package in the FMRIB's Software Library (FSL) [15].

#### 2.3.2 Rigid registration, distortion correction, and artifacts rejection

Five b0 images per DKI dataset were acquired in this study. The b0 image was selected as the reference one by one. Motion-corrupted b0 images were excluded if the normalized LPCCs were smaller than the threshold. The remaining b0 images were averaged serving as the reference image for rigid registration and distortion correction. Rigid registration was performed on FSL [15]. Distortion was corrected by using Automated Image Registration (AIR5.2.5) [16]. If the normalized LPCC between a DWI and the reference was smaller than the threshold, the DWI would be rejected. In the automated rejection method based on LPCC, size of the sub-window was 8×8 pixels. LPCCs were normalized by the maximum of coefficients. The threshold was set to be the standard deviation by a factor of 3 from the average value of the normalized LPCCs in 25 gradient directions per nonzero b value.

#### 2.3.3 Spatial smoothing and tensor estimation

The DWIs after the artifacts rejection were smoothed by using a Gaussian kernel [14]. Diffusion and kurtosis tensors were estimated by using constrained linear least squares (CLLS) [14]. Mean kurtosis (MK), mean diffusivity (MD), and fractional anisotropy (FA) were derived from diffusion and kurtosis tensors [3], [17], [18].

Artifacts rejection and tensor estimation programs were implemented in MATLAB version 7.11.0 (Math Works, Natick, MA, USA).

### 2.4 Development and debugging of the proposed workflow

The datasets of 10 subjects were randomly selected as the experimental data for the development and debugging of the proposed post-processing workflow.

#### 2.4.1 Comparison between NCC and LPCC

In order to evaluate the performance of LPCC and NCC in detecting artifacts, the relative difference between artifacts and artifact-free DWIs was calculated with (Coefficient_artifact-free_−Coefficient_artifact_)/Coefficient_artifact-free._ The DWIs from randomly selected DKI datasets were separated into the artifacts and artifact-free groups according to the normalized LPCCs between the DWIs and the reference b0 images. DWIs whose normalized LPCCs were smaller than the threshold were selected as artifacts. To calculate the differences between artifacts and artifact-free images, the DWIs with higher normalized LPCCs and at the same b values were selected as the paired artifact-free images.

#### 2.4.2 Feasibility of artifacts rejection for DKI

The artifacts rejection may lead to the tensor estimation on partial gradient directions or/and the fewer nonzero b values instead of the original data. It may influence the precision of derived parameters. Therefore, the precision of the derived parameter from the datasets of 15∼25 out of 25 gradient directions and 26 combinations of 2∼5 nonzero b values (listed in Table 1) were evaluated by MSE in this study. During the data acquisition, the worst situation was that the head motion occurred consecutively, especially at the late stage of the MRI scan. To simulate this condition, we removed the consecutive gradient directions from the gradient table manually prior to the tensor estimation. It is difficult to determine which b value would be corrupted by motion, 26 stochastic combinations of 2∼5 nonzero b values were investigated (see Table 1).

**Table 1:** Different numbers and combinations of nonzero b values for the DKI tensor estimation.

No.	Nonzero b values (s/mm^2^)
5	500, 1000, 1500, 2000, 2500
4_#1_	500, 1000, 1500, 2500
4_#2_	500, 1000, 2000, 2500
4_#3_	500, 1500,2000, 2500
4_#4_	1000, 1500,2000, 2500
4_#5_	500, 1000, 1500, 2000
3_#1_	500, 1000, 2500
3_#2_	500, 1500, 2500
3_#3_	500, 2000, 2500
3_#4_	1000, 1500, 2500
3_#5_	1000, 2000, 2500
3_#6_	1500,2000, 2500
3_#7_	500, 1000, 2000
3_#8_	500, 1500, 2000
3_#9_	1000, 1500, 2000
3_#10_	500, 1000, 1500
2_#1_	500, 2500
2_#2_	1000, 2500
2_#3_	1500, 2500
2_#4_	2000, 2500
2_#5_	500, 2000
2_#6_	1000, 2000
2_#7_	1500, 2000
2_#8_	500, 1500
2_#9_	1000, 1500
2_#10_	500, 1000

MSE was used to assess the precision of the derived parameters estimated on the datasets of different protocols. MSE was calculated by the following equation:
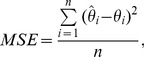
(3)where 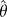, 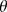 represented the estimated and true values of a parameter respectively, *n* was the number of voxels.

By using the LPCC method, artifact-free experimental datasets of 5 subjects were selected to assess the feasibility of the data rejection for DKI. The parameters estimated on artifact-free datasets with 25 gradient directions and 5 nonzero b values were considered as the reference values. The MSEs were calculated between the reference values and the derived parameters which estimated by the proposed workflow using a different protocol. For a parameter with the additive zero-mean Gaussian noise, minimum of MSE caused by the noise equals the variance of the noise (σ^2^) [19]. The minimum MSE may be larger than σ^2^ when the noise is not additive zero-mean Gaussian noise [19]. σ^2^_L_ may be a strict criterion for MSE to assess different protocols. In this study, σ^2^ was calculated in the image background. The lower bound of σ^2^ (σ^2^_L_) was calculated with mean (σ^2^) − standard deviation (σ^2^). We hypothesized that MSEs of some protocols were smaller than σ^2^_L_. It indicated that influence of the rejected data was less than influence of the noise on the precision of the derived parameters. The smaller MSEs suggested the feasibility for the rejection of motion-corrupted DWIs.

### 2.5 Performance of the proposed workflow in applications

The applicability of the proposed workflow was evaluated by the image quality and the regional quantitative analysis on 36 DKI datasets. The DKI datasets were divided into two groups by using the LPCC method: the control group (n = 18) and the artifacts group (n = 18). The performance of the proposed workflow was compared with that of the conventional post-processing workflow in both the control and the artifacts groups. In the control group, there were no obvious motion artifacts in the DKI datasets. In the artifacts group, the artifacts rejection was performed by using the automated method in the proposed workflow. To verify the feasibility of the artifacts rejection before the DKI tensor estimation, the data of the same gradient directions and/or b values rejected in the artifacts datasets were also excluded manually in the control datasets during our proposed post-processing workflow. For the regional measurement of the derived parameter maps, main parts of white matter were selected as regions of interest (ROIs) by defining the threshold (0.15∼0.3) in an axial FA map.

### 2.6 Statistical analysis

In current study, the Wilcoxon Signed Rank Test was used for comparing the performance in detecting artifacts between NCC and LPCC, and the regional DKI parameter values between the conventional workflow and our proposed workflow. P values less than 0.05 were considered significant. The statistical analysis was performed in SPSS version 13.0 (SPSS Inc., Chicago, IL, USA).

## Results

### 3.1 Development and debugging of the proposed workflow

**Figure 2:**
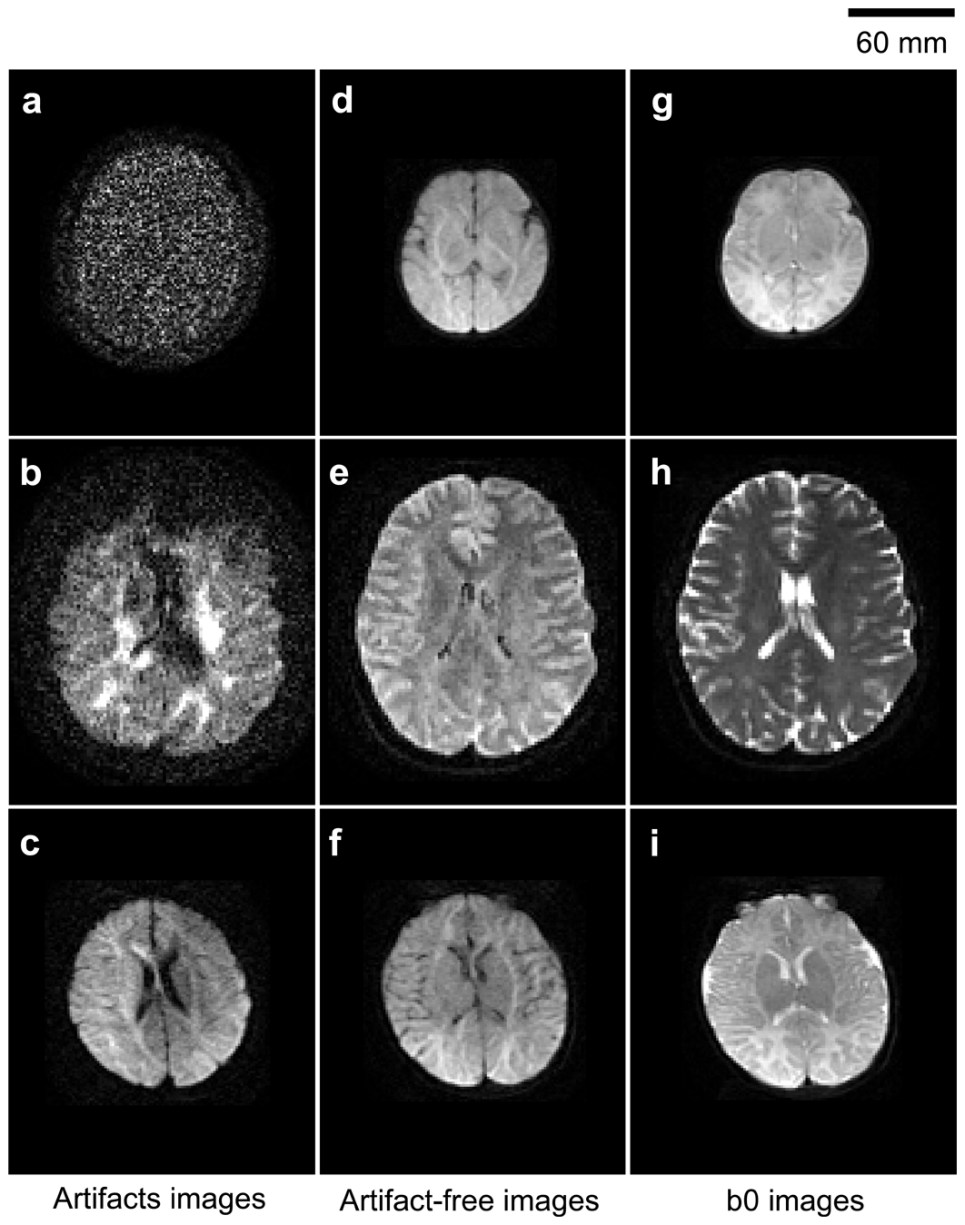
Illustrations of motion artifacts, artifact-free DWIs, and b0 images. Motion artifacts included (a) complete signal loss, (b) local signal loss, and (c) mismatching.

In this study, 10 DKI datasets were randomly selected for debugging the proposed workflow. Thirty motion-corrupted DWIs were found by the visual inspection, while 33 DWIs were rejected according to the normalized LPCC less than the threshold. In Figure 2, the typical motion artifacts, artifact-free DWIs, and b0 images from raw DKI datasets were shown. In the motion-artifact images, Figure 2a demonstrated the complete signal loss caused by sudden head motion with large amplitude. Figure 2b revealed the local signal loss and Figure 2c exhibited the mismatching between slices due to the involuntary motion, which were the frequent artifacts in the DKI datasets.

#### 3.1.1 Comparison between NCC and LPCC

According to the above 33 artifacts images extracted by LPCC method, 33 paired artifact-free images were selected from above 10 DKI datasets. In order to compare the performance of NCC and LPCC in detecting motion artifacts, correlation coefficients between the DWIs and the reference b0 images were obtained. LPCCs and NCCs were normalized by the maximum of coefficients in 25 directions per nonzero b value. As listed in Table 2, normalized LPCCs were lower than normalized NCCs for both artifacts and artifact-free images (p<0.05). With regard to the differences between artifacts and artifact-free images, the relative difference of normalized LPCC was larger than that of normalized NCC (p<0.05). The results indicated that LPCC was more sensitive to detect artifacts than the conventional correlation coefficients.

**Table 2:** Comparison between the normalized NCC and normalized LPCC.

	Nomalized NCC	Normalized LPCC	p value
Artifact-free images (n = 33)	0.98±0.01	0.94±0.04	1.33×10^−5^
Artifacts images (n = 33)	0.84±0.12	0.68±0.14	5.30×10^−7^
Relative difference (n = 33)	0.15±0.12	0.27±0.14	5.32×10^−7^

Note: NCC: normalized correlation coefficient; LPCC: local Pearson correlation coefficient with sub-window of 8×8 pixels; relative difference  =  (Coefficient_artifact-free_ − Coefficient_artifact_)/Coefficient_artifact-free_.

#### 3.1.2 Feasibility of DKI artifacts rejection

**Figure 3:**
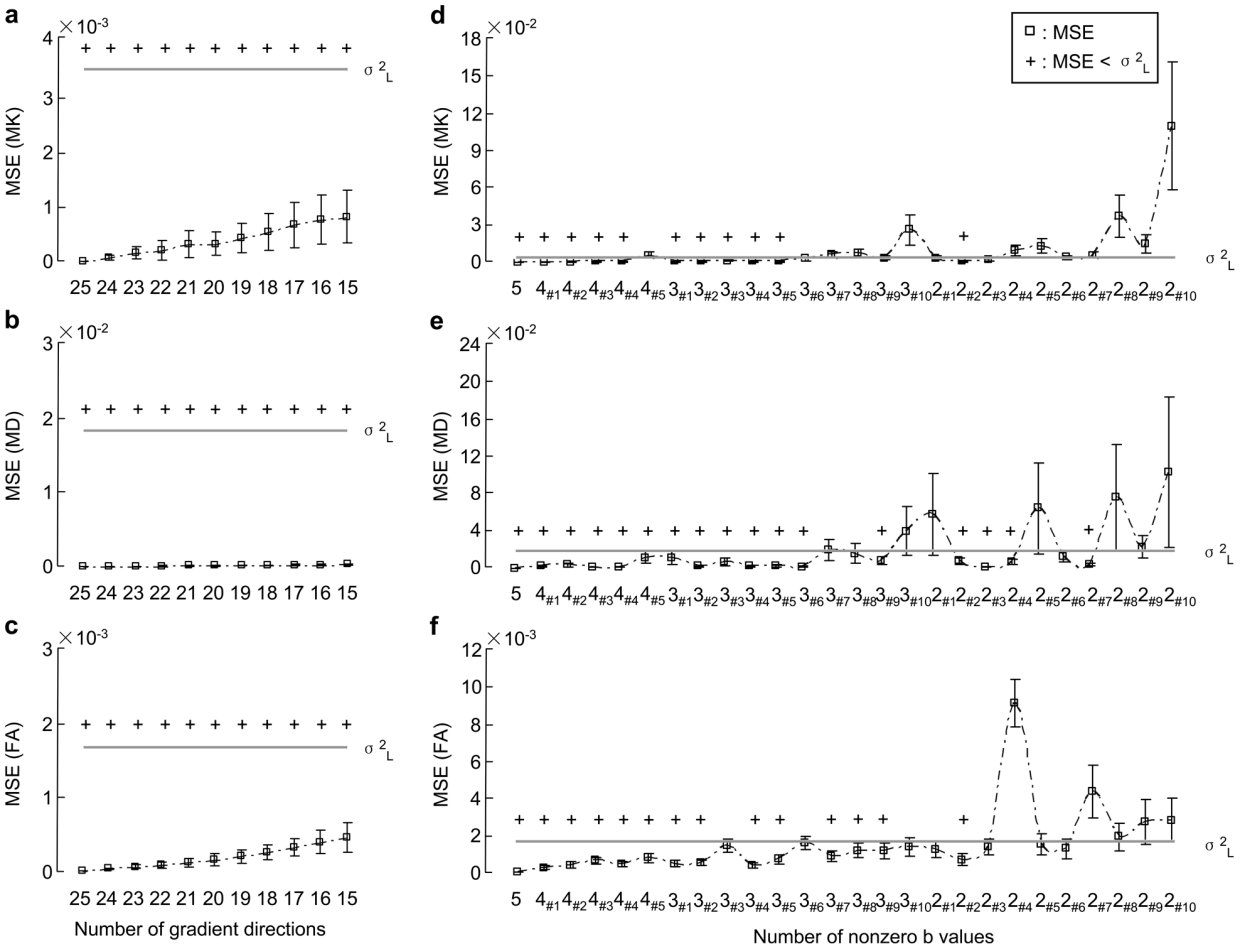
MSEs of MK, MD, and FA estimated on the datasets of the different protocols. The derived parameters were estimated by using the proposed workflow. The protocols included (a∼c) 15∼25 out of 25 gradient directions, and (d∼f) 2∼5 nonzero b values (the combinations of nonzero b values were listed in Table 1). Scatter plots and error bars were the inter-subject averages and standard deviations of MSEs for artifact-free datasets (n = 5), respectively. The cross (+) represented that MSE was less than the low bound of σ^2^ (σ^2^_L_), where σ^2^_L_ =  mean (σ^2^) – standard deviation (σ^2^).

The removal of artifacts may cause that the DKI parameters were derived from the partial gradient directions or/and the fewer nonzero b values instead of the original data. It may influence the precision of derived parameters. We evaluated the precision by using the MSE in the artifact-free experimental datasets of 5 subjects. According to the reference values from the original data of 25 gradient directions and 5 nonzero b values, the MSEs of MK, MD, and FA increased when the data in some gradient directions were excluded (Figure 3a∼3c). However, all of the MSEs were smaller than σ^2^_L_ by using our proposed workflow in this study. It indicated that the rejected data in some gradient directions had little influence on the estimation precision of the derived parameter. Moreover, Figure 3d∼3f showed the MSEs of the estimated parameters on the datasets from the 26 combinations of nonzero b values (listed in Table 1). The MSEs of 11 combinations for MK, 17 combinations for MD, and 14 combinations for FA were smaller than σ^2^_L_ of them. The minimal set of nonzero b values for DKI tensor estimation was the combination of 1000 s/mm^2^ and 2500 s/mm^2^. The set of 500, 1500, and 2500 s/mm^2^ may be also an alternative combination for the tensor estimation.

### 3.2 Performance of the proposed workflow in applications

To examine the performance of our proposed workflow, 36 DKI datasets including 18 motion-corrupted and 18 artifacts-free datasets were processed by using the conventional and the proposed workflows in this study.

#### 3.2.1 Automated artifacts rejection

**Figure 4:**
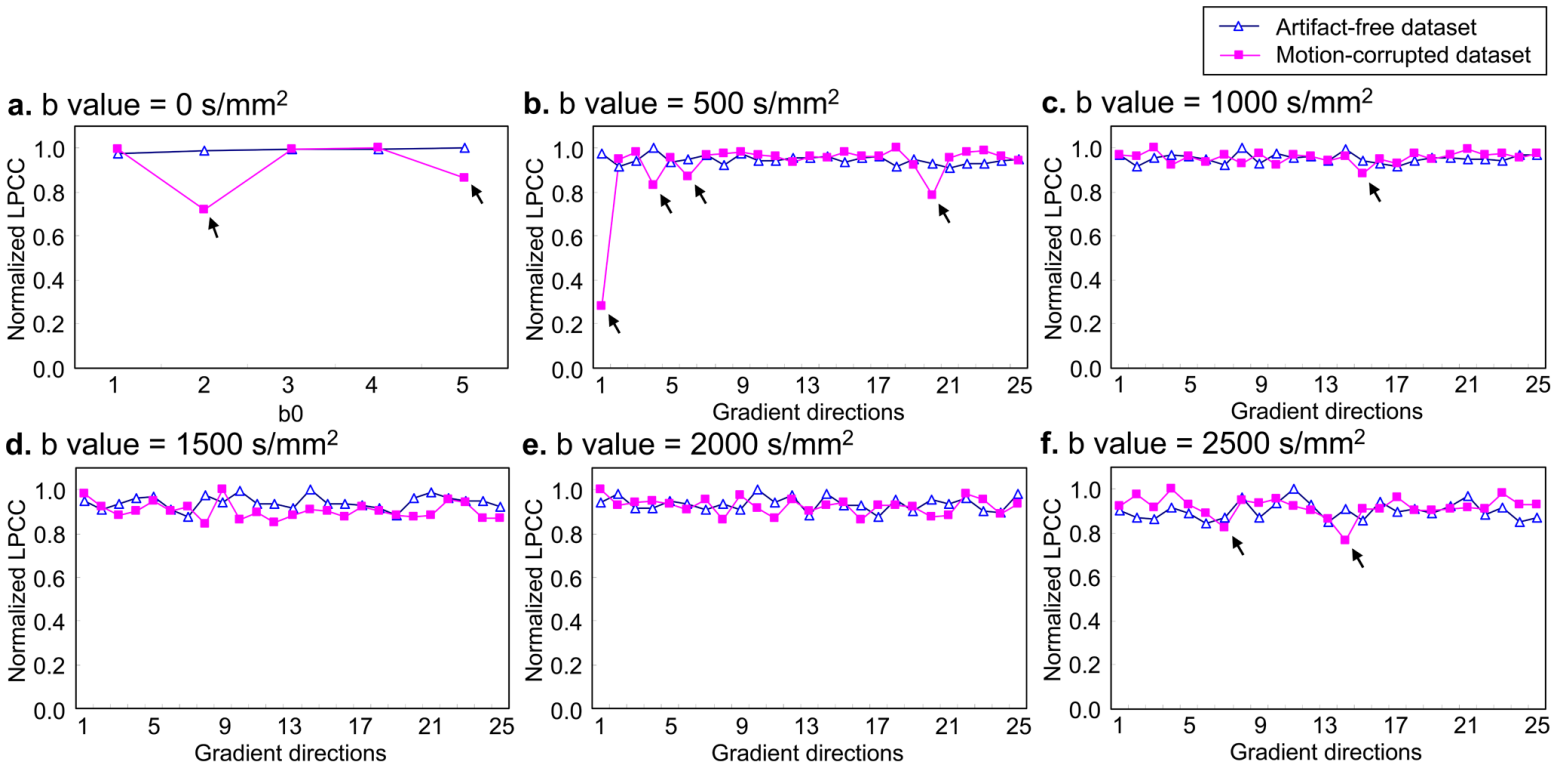
The normalized LPCCs for artifact-free and motion-corrupted datasets. LPCCs were normalized by the maximum of the coefficients. The images whose normalized LPCCs were smaller than the threshold were rejected (the pink diamonds marked by the black arrowheads).

The performance of the automated artifacts rejection method was shown in Figure 4. The fluctuation of the normalized LPCCs in different gradient directions was small in the artifact-free data (the blue triangles in Figure 4). The artifacts (the pink diamonds marked by the black arrowheads in Figure 4) could be detected and rejected easily by our proposed method.

#### 3.2.2 Comparison between the conventional and the proposed workflows

**Figure 5:**
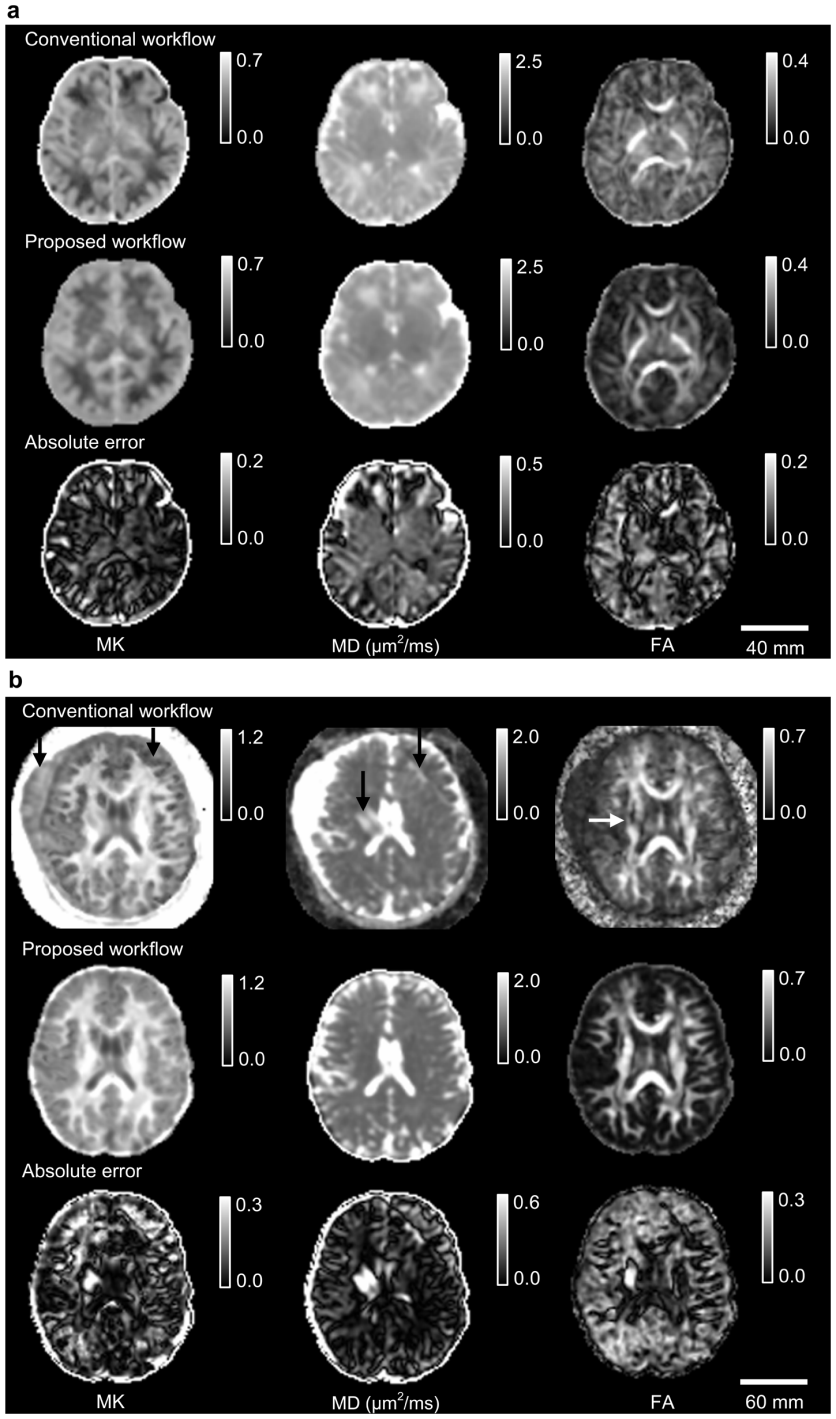
DKI parameter maps estimated by using the conventional and the proposed workflows. Parameter maps included MK, MD, and FA of (a) a neonatal dataset with the signal loss and (b) an adult dataset from the patient of essential tremor with both the signal loss and mismatching. Absolute errors were mapped by the differences of the derived parameters between the conventional workflow and the proposed workflow.

In the Figure 5, the representative parameter maps were derived by using both the conventional and the proposed workflows. In Figure 5a, compared with our proposed workflow, the scattered signal loss exhibited more obviously in the maps of MK and FA derived by using the conventional workflow. In Figure 5b, the artifacts caused by the inter-volume misalignment were visible in all the derived parameter maps by using the conventional workflow (Figure 5b arrowheads). But our proposed workflow removed all artifacts from the DKI parameter maps and demonstrated its robust performance. Maps of the absolute errors in Figure 5 showed that motion artifacts caused evident biases on the derived parameters. Therefore, the rejection of motion artifacts is necessary for the tensor estimation. Our proposed workflow can improve the image quality of DKI derived parameters.

**Figure 6:**
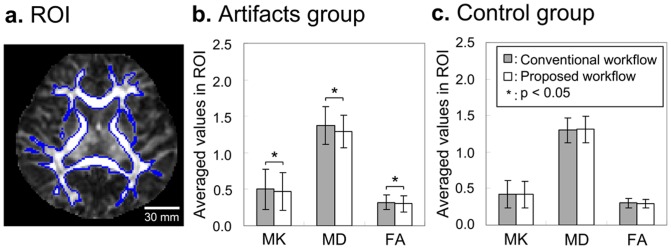
Comparison of ROI measurements between the conventional workflow and the proposed workflow. (a) The ROI of main white matter was overlapped on the FA map. Averaged values of MK, MD in µm^2^/ms, and FA in the ROI were estimated by using both the conventional and the proposed workflows in (b) artifacts group and (c) control group. Error bars were the inter-subject standard deviation in each group (n = 18 per group). The asterisk (*) indicated the significant difference with p<0.05.

In Figure 6, the regional averaged values of DKI parameters were estimated by using both the conventional and the proposed workflows. As shown in Figure 6b, the differences of MK, MD, and FA in ROIs of the main white matter between two workflows were significant (p<0.05) in the artifacts group (n = 18). However, as shown in Figure 6c, no significant differences were found in the artifact-free control group (n = 18), though the data of the same gradient directions or/and nonzero b values were rejected as well. The results clearly demonstrated the robustness of the proposed post-processing workflow in eliminating the motion artifacts influence on the derived parameters and without the effect on artifact-free datasets.

## Discussion

### 4.1 Automatically detecting artifacts: NCC versus LPCC

Motion artifacts limited the application of DKI. This study proposed a robust post-processing workflow to solve this problem. As an efficient artifacts rejection method for DTI quality control, NCC was calculated based on the global information of images [9]. Local artifacts in DWIs may be drowned by the neighboring valid signals during the artifacts detection by using NCC. In human brain DWIs, the background and tissue regions contained different information for the artifacts detection. Therefore an image was divided into several sub-regions in this study. LPCC was used to detect artifacts instead of the conventional correlation coefficient. To detect artifacts from DWIs, LPCC was more sensitive than NCC (Table 2). Different from the combination of LBP and 2D PLS [5], LPCC was obtained directly from the local image intensity. The correlation between two images based on the signal intensity or brightness is a simple method to characterize the agreement between images [13]. To extract local textures, like LBP, in thousands of DWIs in DKI datasets was time consuming. The LPCC rejection method required 0.0575 seconds per slice on a personal computer, which was a fraction of the computational time required by the rejection method of the combination of LBP and 2D PLS. LPCC may be an alternative method for the detection and rejection of artifacts in DKI datasets.

### 4.2 Feasibility of artifacts rejection from DKI datasets

The precision of the DKI derived parameters may be influenced by the removal of artifacts in a number of gradient directions. In current study, MSE was used to evaluate the precision. In theory, 2 nonzero b values and 15 gradient directions per nonzero b value can be used to estimate the 21 independent elements in diffusion and kurtosis tensors [2], [14], [20]. The proposed gradients direction number was 20 for the clinical use [21]. Considering the data in some gradient directions that may be corrupted by head motion, 25 gradient directions were acquired in this study. This study investigated the precision of the derived parameters from the datasets of 15∼25 out of 25 gradient directions. Our results demonstrated that MSEs of the derived parameters in 15 gradient directions were higher than those in 20 gradient directions and more, which is consistent with a previous study [21]. But all the MSEs were smaller than σ^2^_L_ (Figure 3). This result in our study ensured that tensors of DKI could still be estimated if a remaining dataset would be provided with at least 15 gradient directions. In the same way, if the gradient direction number of DWIs in one nonzero b value was less than 15, all the DWIs in this nonzero b value should be rejected.

Compared with the conventional DTI model, the diffusivities in DKI are less dependent on the b values [22]. However, the kurtosis tensor is estimated based on both the first-order and the second-order terms in the DKI model. Both low and high b values were necessary for the DKI tensor estimation. In current study, MSEs of 2 nonzero b values (1000 and 2500 s/mm^2^) were smaller than σ^2^_L_ (Figure 3), which confirmed the feasibility of artifacts rejection for DKI.

In sum, the precision of the tensor estimation in DKI depended on the distribution of the reserved nonzero b values. The reserved data after the artifacts rejection should contain the minimum effective set of the gradient directions and b values for the tensor estimation.

### 4.3 Limitations and potential applications

Despite that the proposed workflow performed well to improve the image quality and the accuracy of quantitative analysis on motion-corrupted DKI datasets, there were some limitations. The main weakness was that LPCC based on correlation of image intensity could not detect the images of good tissue contrast with hyper-intensity or hypo-intensity. Another aspect of LPCC remained to be improved was the weighting coefficients of sub-regions. In this study, sub-regions contained tissues were weighted equally. An optimized framework for the weighting coefficients may be more reasonable for calculating LPCC. The rejection criterion for the normalized LPCCs was set to be the standard deviation by a factor of 3 from the average value. The factor for the threshold remained to be calibrated on large scale datasets. Moreover, the problem of cerebrospinal fluid (CSF) partial volume effect in DKI [3] was not solved in our workflow.

Motion artifacts were common in diffusion MRI techniques. Compared with DTI, both the HARDI and DKI required a longer scan time and held DWIs of relatively high b values. The proposed workflow may be also suitable for the detection and rejection of the motion artifacts for other diffusion MRI techniques, like the HARDI. Since the signal to noise ratio (SNR) of the image in the high b value was low, the unreliable registration for the data of the very high b value in the HARDI datasets [1] was a challenge for the application of our workflow. The future work will focus on the improvement of the proposed post-processing workflow.

In conclusion, the proposed post-processing workflow was reliable to improve the image quality and the measurement precision of the derived parameters on the motion-corrupted DKI datasets. The workflow provided an effective post-processing method for the clinical applications of DKI in the subjects with involuntary movements.
